# Smart homes energy management: Optimal multi-objective appliance scheduling model considering electrical energy storage and renewable energy resources

**DOI:** 10.1016/j.heliyon.2025.e42417

**Published:** 2025-01-31

**Authors:** Moslem Dehghani, Seyyed Mohammad Bornapour

**Affiliations:** Electrical Engineering Department, Faculty of Engineering, Yasouj University, Yasouj, Iran

**Keywords:** Home energy management systems, Electricity selling, Photovoltaic, Wind turbine, Energy storage systems, Biogeography-based optimization algorithm

## Abstract

As smart homes (SHs) integrate into distribution systems, microgrid scheduling has become increasingly important because of their schedulable loads that reduce peak loads. Accordingly, a multi-objective optimization approach is presented for SH energy management (SHEM) and demand response (DR) programs with 30-min time slots. Time-of-use tariffs are used in the suggested scheme, and the primary goal is to minimize the daily bills and peak-to-average ratio (PAR), simultaneously. This scheme includes flexible and fixed home appliances. Here, the SHEM system consists of photovoltaic and wind turbine systems in combination with an electrical energy storage (EES) system to provide optimum peak load performance at peak times, based on the discharging and charging mechanism. Also, in the proposed mathematical formulation, the bought and selling energy is considered during the day. An improved Biogeography-based optimization algorithm (IBBO) is used to solve the multi-objective problem. The first step is to develop the equations for general electrical appliances of particular SH consumers, and then minimize the mentioned two objectives. Based on the outcomes under different scenarios such as different sizes of renewable energy resources, various charging/discharging rates, and different selling electricity tariff ratios, PAR and operational costs are reduced, and the electricity is sold to upstream. Moreover, simulations show that the suggested scheme produces the optimal outcomes, in which both objectives are near their optimal levels, as shown in the Pareto Front of the optimal solutions. The maximum standard deviation of total objective function between all cases for IBBO, gray wolf optimizer (GWO), and whale optimization algorithm (WOA) are 6.55, 17.22, and 24.87, respectively, which show the robustness of IBBO in finding the best solution in comparisons of other algorithms. Also, the average solution of IBBO is lower than GWO, and WOA, which shows the performance and superiority of IBBO in finding the best solution.

## Highlights

• Appliances, energy storage, PV, and wind turbine are scheduled simultaneously.
• Both economic (costs) and technical factors (PAR) are optimized simultaneously.
• An improved Biogeography-based optimization algorithm is used to solve the problem.
• Various sizes of RER, and diverse selling electricity tariff ratios are considered.
• Different charging/discharging rates of ESS are investigated.

## 1 Introduction

Climate change and global warming continue to worsen, leading to disasters like sea ice melt, severe floods, destructive hurricanes, etc. $CO_{2}$ emissions produced by burning fossil fuels for energy are one of the major contributors to global warming. Several research projects are being conducted to address this issue, such as developing renewable energy resources (RERs) instead of fossil fuels and integrating RERs with energy storage systems (ESS).

A smart grid (SG) is designed to replace the current power grid which no longer supports consumers' changing demands. With the SG, the conventional electrical power system is improved through the use of modern communication technology. SG can reduce carbon dioxide and greenhouse gas emissions, and mitigate climate change by integrating RER and ESS [1]. It has two important advantages over conventional grids: advanced metering infrastructure (AMI) and demand side management (DSM).

Energy measurement and information collection are the main functions of AMI. The system includes smart meters as well as information and communication technology (ICT). As a result of ICT, SG can update users on changes in energy costs, or problems resulting from equipment or a natural disaster. Moreover, the smart meter transmits energy usage data to electricity operators for monitoring and analyzing real-time data and making real-time decisions.

SG's energy management relies heavily on DSM. There are a variety of functions provided by DSM, including control of the electricity market, management of energy resources, construction of infrastructure, and decentralized energy resource management [2,3]. Various concepts are involved in DSM, including demand response (DR) and energy optimization. Utility and consumer demand are balanced under DSM, whereas consumer demand is concentrated only under DR. In the DR, programs assist customers in reducing short-term energy demands based on cost signals generated by the market or triggered by grid operators [4]. DR focuses on adjusting the power demand more than the supply. The study, despite this, indicates that scholars consider DSMs and DRs equivalent [5].

Generally, SG's DSM and DR functions motivate users to use RERs to power their loads, particularly during peak periods, so they are less dependent on utility companies. Secondly, users are encouraged to move their energy usage from peak to off-peak times by shifting their home loads [4,6]. As a result of the above goals, users are also able to decrease electric bills and energy usage during peak periods. Furthermore, as electric mobile equipment, including electric vehicles (EVs), and RER technology continue to develop, more and more users are selling excess energy on the market. A buyer can be an electric car, another house, or even an energy provider. A home energy management system (HEMS) would be needed at the consumer end for these objectives to be achieved. In addition to controlling and optimizing RER, ESS, and household devices, HEMS can help users engage in DSM-related activities.

There have been numerous papers proposing algorithms for improving the performance of household equipment, including linear programming (LP) [7], mixed integer LP (MILP) [8,9], mixed integer nonlinear programming (MINLP) [10,11], dynamic programming (DP) [12], or convex programming (CP) [13]. The disadvantage of such methods is that their convergence rate is extremely slow when applied to many variables, and sometimes they are incapable of handling multiple devices [4,14]. HEMS employs meta-heuristic algorithms to overcome these issues. There are several meta-heuristic algorithms commonly employed in optimization, such as particle swarm optimization (PSO), binary PSO, genetic algorithm (GA), wind-driven optimization, bacterial foraging optimizations, and the Jaya algorithm [4,[14], [15], [16]].

A Time-of-use (TOU)-driven limited memory algorithm was used in Ref. [17] to optimize the scheduling of household devices, with photovoltaic **(**PV) and ESS. Ref [18] employed an advanced adaptive PSO (AAPSO) to optimize the operating strategy for a hybrid PV battery in HEMS. As a result, the yearly costs were decreased by 28 % by applying the AAPSO to solve a MILP problem. A HEMS with a stationary storage system was implemented in Ref. [19] using a TOU-driven multi-objective linear approach. In addition to reducing household energy costs, the proposed scheme could additionally reduce the peak load demand for the system. Ref [20] developed an HEMS by considering intermittent renewable power generation in domestic homes. It is important to note that the outcomes are case-specific and primarily affected by the case studies examined. Despite this, the suggested approach reduced energy costs by 42 %. Ref [21] solved the optimal appliance scheduling problem as a single-objective optimization problem using binary gray wolf optimization. Ref [22] presented an effective HEMS that reduces energy costs and peak load demands while reducing emissions. The model used DR programs, or real-time pricing (RTP), which helped reduce energy demand in low-price periods.

Ref [10] presented a home automation/energy management system (HAEMS) incorporating ESS. An objective function consisting of consumer satisfaction, the cost of energy, as well as thermal comfort, was optimized in the study. It utilized RTP pricing in its system. Specifically, GAMS software with Cplex/Dicopt solvers was used in the study for optimization. A naive, normal, and smart scenario was considered for their HAEMS. Based on the simulations, the HAEMS enhanced the mixed objective function up to 55 % and 25 % in the smart case as compared with naive and normal for hot weather, and up to 63 % and 38 % in cold weather. The study excluded both RER and peak-to-average ratio (PAR).

Ref [9] examined smart home (SH) optimization and energy management. In addition to ESS and EVs, wind and solar energy were integrated to optimize energy usage. Energy sales were likewise taken into account. Three case studies were performed: one day, four days, and seven days. The case studies were evaluated using MILP and heuristic algorithms. Due to the absence of an AMI in their system, electricity operators were unable to provide them with information. The system only forecasts the load for the day and does not describe devices in the home. No specific plan was provided regarding household devices. DSM does not apply to system. A second limitation is that it merely considers reducing energy costs while not considering PAR.

Ref [12] examined a residential smart energy management system (RSEMS) that integrates ESS and RER. A hybrid objective function is used to optimize thermal comfort, the cost of energy, and customer comfort at the same time. Optimizations were carried out using MATLAB and GAMS. 2 case studies were studied to compare the RSEMS to traditional EMS: on a hot winter day and on a cold summer day. As a result of their RSEMS, hybrid objective function improved by up to 29 % in hot weather and by up to 33 % in cold weather. Unfortunately, the study did not consider PAR. Activities related to energy sales and the use of main grid energy for low prices are discussed, but not in-depth, in Refs. [10,12]. A schedule of sales operations during a particular period was not provided. Furthermore, no consideration was given to a sale price. It is impossible to apply their designs if the sale cost falls below the main grid cost.

Ref [7] examined an optimal energy management system for reducing power costs. The paper used the ESS and plug-in hybrid EV (PREV) to measure electricity and calculate optimized values. ESS discharges electricity for home use during high demands, and the PREV supplies power to ESS during low demands. The optimization problem was solved using LP. The study did not include RERs. Ref [23] examined an ontology-based multi-agent energy management system (MAS). By using the model, a microgrid (MG) system for houses or buildings could be monitored and controlled optimally. Various agents have been used at home, such as EMS, to optimize operational strategies by cooperating. Additionally, the model includes practical agents, including the central coordinator agent that collects and shares data, and the battery bank agent (BBA) that compensates for real-time power imbalances in household MGs. As well as storing excess energy, the BBA provided energy back to the MG. Energy could be bought or sold by the BBA from the utility. 3 case studies were tested: naive, normal, and smart, each with a unique period (weekends and weekdays), climate (cold and hot), and price schemes like: TOU, and RTP. Simulation results showed that the suggested MAS could decrease the operational price of the system and meet the demands of the customers in a variety of weather, periods, and price plans. MAS, however, considered the entire MG, not just one house.

Table 1 shows a comparison between different published papers in the SHEM area [[24], [25], [26], [27], [28], [29], [30], [31]]. As can be seen, wind turbine (WT) is not considered in most recent works and also PAR which is a main technical factor in operation to balance the loads in the system is not considered in some works [24,27,28,30,31].

**Table 1:** Comparison between various papers in the literature.

Ref.	Method	Objective Functions	PV	WT	ESS	Description
[24]	Whale optimization algorithm with hybrid reverse learning competitive strategies	Electric cost, and carbon emissions	√	–	√	PAR is not considered.
[25]	Adaptive Coati Optimization algorithm	Electric cost, and PAR	√	–	√	The system is not considered the selling energy to upstream.
[26]	Binary Particle Swarm Optimization algorithm	Electricity cost	√	√	√	The problem is optimized in single-objective mode and only electricity cost is investigated. Uncertainty is considered.
[27]	Constructive Heuristic, improved by the Multi-Start Algorithm	Electricity cost, and comfort index	√	–	–	PAR is not considered.
[28]	Whale Optimization Algorithm	Electricity cost	√	–	√	PAR is not considered.
[29]	Gurobi Solver	Electricity cost	√	–	√	The problem is optimized in single-objective mode and only electricity cost is investigated. Uncertainties are considered.
[30]	NSGA-III	Electricity cost, and users' comfort	√	–	√	PAR is not considered. Photovoltaic/Thermal is considered to optimize both electrical and thermal loads.
[31]	Hybrid robust-stochastic (HRS) optimization approach	Electricity cost, and users' comfort	√	–	√	PAR is not considered. Uncertainties in PV and RTP are considered by applying robust optimization schemes.
This paper	Improved Biogeography-based optimization algorithm	Electricity cost, and PAR	√	√	√	The problem is solved under different sizes of RERs, various charging/discharging amounts of ESS, and diverse selling electricity tariff ratios.

Based on the above research, most of the research only considers the electricity cost, and the PAR as a technical factor is not investigated. Furthermore, they used weight ratio to make the multi-objective problems into a single objective problem, but in this paper by using the Parto front, a set of solutions is obtained for the proposed multi-objective problem. Also, in this paper, PV, WT, and ESS systems are considered in the suggested mathematical formulas, and the amount of selling electricity is determined in each time slot (30 min). As most of the literature, used 1 h as the time slot, and it is big for appliance scheduling, the 30-min time slot is considered in this study. By using the suggested energy management, the ESS is charged in low price time and sold the electricity to the upstream in high price times. In conclusion, despite the fact that multi-objective energy management is discussed in a few papers, this paper provides novel contributions including:

• In SH, appliances PV, WT, and ESS have been optimized simultaneously with 30-min time slots, for greater efficiency.
• The SH's operating time is calculated under different scenarios such as different sizes of RERs, various charging/discharging rates of ESS, and also different selling electricity tariff ratios.
• The multi-objective optimization model is presented for the SHEM system considering both economic (total costs) and technical factors (PAR).
• SHEM system is considered with ESSs, PVs, and WTs, simultaneously and also selling electricity to the upstream.
• An improved Biogeography-based optimization (BBO) algorithm with Pareto Front is used to solve the multi-objective optimization problem.

Following are the sections of the study. The SH system is described in Part 2. Part 3 describes the suggested DSM scheme with objective functions. The BBO algorithm is explained in Part 4. A numerical analysis is presented in Part 5 as well as a discussion of relevant issues. The conclusion can be found in Part 6.

## 2 HEMS

In a HEMS, smart devices are monitored, controlled, and automated effectively in the shortest amount of time possible [32]. In addition to providing energy arbitrage, DR and self-usage reduce electricity bills.

**Fig. 1:**
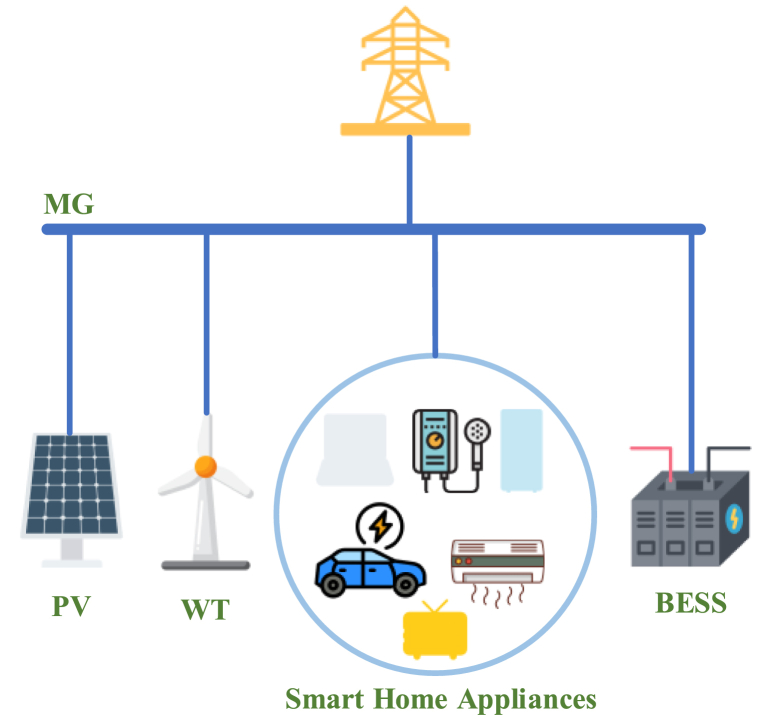
Standard HEMS structure.

Fig. 1 illustrates the structure of an energy management system for a home. WTs, solar panels, and ESS are all distributed energy resources. In the EES system, a portion of the power created by RERs is stored during off-peak periods and discharged at peak times, thereby reducing operational expenses and enhancing the flexibility of the grid.

Through AC buses, the MG system was interconnected with the grid and operated as a power distribution system. Residential loads were scheduled using the DSM method. Hourly load demands were met with renewable energy produced by RERs.

## 3 Problem formulation

Every SH's HEMS optimizes the operating time of its appliances based on production and consumption energy balance restrictions. Every period (t) must evaluate this restriction as shown by Eq. (1).

(1) $$P_{MG2SH}\left(t\right)+P_{PV}\left(t\right)+P_{WT}\left(t\right)+P_{Bat}^{dis}\left(t\right)=P_{SH2MG}\left(t\right)+P_{APP}\left(t\right)+P_{Bat}^{ch}\left(t\right)$$

Where $P_{MG2SH}\left(t\right)$ is purchased energy from the smart MG (SMG). $P_{PV}\left(t\right)$, and $P_{WT}\left(t\right)$ are the produced power of household PV and WT. $P_{Bat}^{dis}\left(t\right)$ is the power of the ESS during discharging. $P_{SH2MG}\left(t\right)$ is the selling energy to the SMG. $P_{APP}\left(t\right)$ is the overall demand for house devices. $P_{Bat}^{ch}\left(t\right)$ is the power of the ESS during charging. Here, SHs' user and producer appliances have been numerically analyzed.

### 3.1 Devices

As part of an SH, smart devices are an essential component. The welfare of consumers can be improved by controlling and managing devices. There are two types of devices in SHs: shiftable devices and fixed devices.

It is impossible to manage fixed appliances such as refrigerators and lights. Thus, the user determines when the fixed appliances start and end. There is also a group of appliances called shiftable devices, which can be managed. HEMS can schedule the usage time of shiftable appliances. The HEMS determines shiftable devices' operating times for improved performance. Consequently, every appliance's per-day state vector is defined using Eq. (2). Here, UT(t) equals 1 or 0 depending on whether device k is on or off during time slot t.

(2) $${UT}_{k}=\left[{UT}_{1},{UT}_{2},\ldots ,{UT}_{T-1},{UT}_{T}\right]$$

It is a limitation that each appliance will be run during the daytime; details appear in Eq. (3). Various operation times apply to appliances. For uninterrupted appliances, whenever a device is started, it must run continuously with no interruptions. For interrupted appliances, whenever a device is started, it can be interrupted and also it should meet the operational time. A description of this limitation can be found in Eq. (4). Accordingly, appliances should be started according to Eq. (5).

(3) $$\sum_{t=1}^{n_{ts}}UT_{kt}\geq 1$$

(4) $$\sum_{t=\tau_{s}}^{\tau_{e}}UT_{kt}=OP_{k}$$

(5) $$\tau_{s}\leq n_{ts}-OP_{k}+1$$

Here, $n_{ts}$ shows the number of time slots for a day, $\tau_{s}$ is the start time of device k, $\tau_{e}$ is the end time of device k, $OP_{k}$ shows the sufficient number of operational timing intervals for device k.

Appliance usage is assumed to be constant throughout the operation timeframe. The device's power demand pattern throughout its operating time is assumed to be fixed.

### 3.2 Photovoltaic panel

A PV system can produce clean electricity from the sun's energy by utilizing unutilized spaces like rooftops and backyards. The SH is less dependent on the SMG when PV units are used. Solar panels' performance and area determine their power, as does solar irradiance. Due to the stochastic nature of illumination intensity, the PV unit's output power is unpredictable. Consequently, PV units should be modeled using a stochastic model.

The beta probability density function (PDF) shows the solar irradiance according to the historical data at each period of the day [33]. The PDF of solar irradiance at each period t is calculated based on Eq. (6) in the following:

(6) $$f_{B}\left(\emptyset_{t}\right)=\left\{ \begin{array}{c} \frac{\Gamma \left(\alpha_{t}+\beta_{t}\right)}{\Gamma \left(\alpha_{t}\right)\Gamma \left(\beta_{t}\right)}*\emptyset_{t}^{\alpha -1}*{\emptyset_{t}}^{\beta -1}\;\left(\begin{array}{l} 0\leq \emptyset_{t}\leq 1\\ \alpha_{t},\beta_{t}\geq 0 \end{array}\right)\\ 0\;Otherwise \end{array}\right.$$

Where $f_{B}\left(\emptyset_{t}\right)$ defines the Beta PDF of $\emptyset_{t}$ that shows a random variable of solar irradiance $\left(kW/m^{2}\right)$ at period t. $\alpha_{t}$ and $\beta_{t}$ show the Beta PDF parameters that are used to compute the standard deviation ($\sigma_{t}$), and mean ($\mu_{t}$) amounts of solar irradiance $\emptyset_{t}$ by Eq. (7), and Eq. (8):

(7) $$\beta_{t}=\left(1-\mu_{t}\right)*\left(\frac{\mu_{t}*\left(1+\mu_{t}\right)}{{\sigma_{PV,t}}^{2}}-1\right)$$

(8) $$\alpha_{t}=\frac{\mu_{t}*\beta_{t}}{1-\mu_{t}}$$

s shows the discrete states, at each period t, $\lambda_{s,t}$ is the probability of each state, which is computed by Eq. (9) in the following:

(9) $$\lambda_{s}\left(t\right)=\int_{\emptyset_{n-1}}^{\emptyset_{n}}f_{B}\left(\emptyset_{s}\right)*d\emptyset_{s}$$

In which, $\emptyset_{n-1}$ and $\emptyset_{n}$ show the limits of solar irradiance for the $s^{th}$ state. Eq. (10) shows the calculation of the output power of PV at each state as below:

(10) $$P_{PV}\left(\emptyset_{s,t}\right)=\eta_{PV}*S_{PV}*\emptyset_{s,t}$$

Where $\eta_{PV}$ is the efficiency and $S_{PV}$ is the overall area ($m^{2}$) of the PV panel. Finally, the entire anticipated PV output power (kW) over a given period Δt can be computed by Eq. (11):

(11) $$P_{PV}\left(t\right)=\sum_{s\in S}\lambda_{s}\left(t\right)*P_{PV}\left(\emptyset_{s,t}\right)$$

### 3.3 WT

Wind power systems convert wind speed into electricity. In the WT energy system, the output power is influenced by the wind speed every hour [34]. Based on Eqs.(12), (13), (14)), a Weibull probability distribution function can be applied to predict wind speed distribution [35]:

(12) $$f=\frac{\lambda }{\xi }*{\left(\frac{V}{\xi }\right)}^{\lambda -1}*e^{-{\left(\frac{V}{\xi }\right)}^{\lambda }},1\leq \lambda \leq 10$$

(13) $$\lambda ={\left(\sigma_{WT}/\bar{V}\right)}^{-1.086}$$

(14) $$\xi =\frac{\bar{V}}{\Gamma \left(1+\frac{1}{\lambda }\right)}$$

Here λ is the shape factor, ξ is the scale factor, V shows real-time wind speed, $\bar{V}$ shows mean, $\sigma_{WT}$ is the standard deviation. The WT output power can be obtained by Eq. (15) [36,37]:

(15) $$P_{WT}=f\left(V\right)*\left\{ \begin{array}{c} 0,V<V_{I}\;and\;V\geq V_{0}\\ P_{WT}^{R}*\frac{V-V_{I}}{V_{R}-V_{I}},V_{I}\leq V\leq V_{R}\\ P_{WT}^{R},V_{R}\leq V\leq V_{0} \end{array}\right.$$

In which, $P_{WT}$ is the output power from WT output power, $P_{WT}^{R}$ is the WT-rated power, $V_{R}$ is the rated wind speed, $V_{I}$ is the cut-in speed. $V_{O}$ is the cut-out speed.

### 3.4 Battery ESS (BESS)

The discharging and charging of BESS would be obtained by Eq. (16) and Eq. (17), respectively [38,39]:

(16) $$P_{Bat}^{dis,Load}\left(t\right)+P_{Bat}^{dis,MG}\left(t\right)=\eta_{Bat}^{Ch/dis}.P_{Bat}^{dis}\left(t\right),\forall t$$

(17) $$P_{Bat}^{Ch,RER}\left(t\right)+P_{Bat}^{Ch,MG}\left(t\right)=\eta_{Bat}^{Ch/dis}.P_{Bat}^{Ch}\left(t\right),\forall t$$

Here, $P_{Bat}^{dis,Load}\left(t\right)$ is the power transfer from Battery to loads, $P_{Bat}^{dis,MG}\left(t\right)$ is the power transfer from Battery to MG (Selling Energy), $P_{Bat}^{Ch,RER}\left(t\right)$ is the power transfer from RERs to ESS, $P_{Bat}^{Ch,MG}\left(t\right)$ is the power transfer from MG to ESS, $P_{Bat}^{Ch}\left(t\right)$, and $P_{Bat}^{dis}\left(t\right)$ show the charge and discharge power of ESS, respectively, $\eta_{Bat}^{Ch/dis}$ is the ESS discharge efficiency. The BESS can only be in charging mode or discharging mode, hence Eq. (18), and Eq. (19) show the status of charging or discharging of BESS in following:

(18) $$0\leq P_{Bat}^{ch}\left(t\right)\leq R_{Bat}^{ch}.x_{Bat}\left(t\right),\forall t$$

(19) $$0\leq P_{Bat}^{dis}\left(t\right)\leq R_{Bat}^{dis}.\left(1-x_{Bat}\left(t\right)\right),\forall t$$

Here, $R_{Bat}^{ch}$ is the charge rate of ESS, $R_{Bat}^{dis}$ is the discharge rate of ESS, $x_{Bat}\left(t\right)$ shows a binary variable showing the charge and discharge state of the ESS. $x_{Bat}\left(t\right)$ is 1 during the charge state of ESS during time t, if not it equals 0. The energy level of BESS can be modeled by Eqs.(20), (21), (22)) as below:

(20) $$SoE_{Bat}\left(t\right)=SoE_{Bat}^{initial},if\;t=1$$

(21) $$SoE_{Bat}\left(t\right)=SoE_{Bat}\left(t-1\right)+\eta_{Bat}^{ch}.P_{Bat}^{ch}.\Delta t-{\eta_{Bat}^{dis}.P}_{Bat}^{dis}.\Delta t,\forall t$$

(22) $$SoE_{Bat}^{\max}.\left(1-DoD_{Bat}\right)\leq SoE_{Bat}\left(t\right)\leq SoE_{Bat}^{\max},\forall t$$

Here, $SoE_{Bat}\left(t\right)$ shows the mode of energy of ESS, $SoE_{Bat}^{initial}$ is the primary level of ESS during the first time (t=1), $SoE_{Bat}^{\max}$ shows the maximal capacity of ESS, and $DoD_{Bat}$ is the depth of discharge.

As shown in Eq. (16), BESS discharged power equals the combination of energy consumed within the home and selling to the MG. Based on Eq. (18) and Eq. (19), at any one time, the BESS cannot charge or discharge the battery faster than the battery charging rate. Eq. (20) and Eq. (21) describe the state of energy (SoE) in the BESS. According to Eq. (22), the BESS's SoE must not be larger than its maximal battery capacity, and its minimal SoE is determined by its allowable depth of discharge.

### 3.5 Objective functions

The objective of the study is to combine economic and technical indicators in homes connected to an SMG using DSM. Here, the electricity bill ($OF_{1}$) of the SH and the technical index that shows the index of the PAR ($OF_{2}$) demand are considered.

#### 3.5.1 Electricity bill

An SMG's energy bill can be calculated by adding up the electricity bills of SHs. As a result, whenever every SH's energy bill decreases, the SMG's electricity bill decreases the most. In every SH, the HEMS is responsible for selecting the best energy sources and household devices during operation. A SH's electricity demand is met by the HEMS with energy provided by the SMG and home sources. Home energy sources include WT and PV panels. Depending on the amount of solar radiation and the hour, PVs generate variable power. Thus, the HEMS schedules appliance and home energy usage for each hour to reduce the customer's energy bill and SMG's overall energy bill.

Therefore, Eq. (23) calculates the SMG's energy bill according to its income and costs. Here, as the RERs are a part of SH, the cost of energy of RERs is considered zero. $C_{SMG}$ shows the purchased energy cost from the SMG. $I_{SMG}$ shows the income from the sale of the energy to the SMG.

(23) $$OF_{1}=\sum_{t=1}^{T}C_{SMG}\left(t\right)-I_{SMG}\left(t\right)$$

Consumers purchase energy from Distribution Companies at variable prices. Home energy sources (PV and WT) supply part of the electricity demand while SMG purchases the rest.

Eq. (24) calculates the overall consumption of all devices for every SH during the time slot.

(24) $$P_{app}\left(t\right)=\sum_{i=1}^{l}pa_{i}$$

Here, l shows the number of devices, $pa_{i}$ shows every device's energy consumption during time t, and $P_{app}(t$) shows the overall energy consumption of household devices during time t.

The other SH component that influences the purchase of energy would be the ESS. Energy is injected into the network by the ESS during the discharging state. ESS in the charging state, however, raises the SH's energy usage.

According to international scientific consensus, to prevent worsening climate damage, net human-caused carbon dioxide ($CO_{2}$) emissions need to be reduced by about 45 % from 2010 to 2030, and also reach zero by 2050 [40]. Hence, we assumed, the energy cost of WT, and PV are zero to use the maximum energy of RERs. Thus, Eq. (25) calculates the per-hour surplus demand of every SH during time *t* to be met by SG.

(25) $$P_{SH}\left(t\right)=P_{app}\left(t\right)-P_{PV}\left(t\right)-P_{WT}\left(t\right)-P_{ESS}^{dis,Load}\left(t\right)+P_{ESS}^{Ch,MG}\left(t\right)$$

Furthermore, Eq. (26) calculates energy purchase costs from the MG by home *i* each day.

(26) $$C_{SMG}=\left\{ \begin{array}{c} P_{SH}\left(t\right)*Price^{MG}\left(t\right);\;\text{if}\;P_{SH}\left(t\right)>0\\ 0;\;\text{if}\;P_{SH}\left(t\right)\leq 0 \end{array}\right.$$

$Price^{MG}\left(t\right)$ shows the electricity tariff during time *t.* The independent system operator determines the electricity tariff here. In the case of a scheduling period of 30 min, there would be 48 periods in the day ahead (T = 48).

Based on Eq. (26), an SH should purchase energy from the SMG when its devices and ESS (charging mode) consume more energy compared to what its RERs generate. Whenever home energy sources produce more energy than devices consume, SHs can sell energy to the SMG (It is assumed that only the ESS can sell energy), and also the surplus energy produced by RERs after charging the ESS and feeding the SH loads is wasted. Eq. (27) calculates revenue from energy sales to the SMG. Power is sold back to the SMG at a fixed price between the user and the system operator.

(27) $$I_{SMG}=P_{ESS}^{dis,MG}*Price^{sell}\left(t\right)$$

In which, $Price^{sell}\left(t\right)$ shows the energy cost sold back to the SMG during time t and it would be predefined between the user and the system operator. Here, it is supposed the selling cost is a ratio (ρ) of electricity tariff, so the electricity cost objective function is presented by Eq. (28):

(28) $$OF_{1}=\sum_{t=1}^{T}\left(P_{SH}\left(t\right)-\rho P_{ESS}^{dis,MG}\right)*Price^{MG}\left(t\right)$$

#### 3.5.2 PAR

In spite of the fact that MG peak demand happens only a few times throughout the day, it is important that the MG provider is prepared to meet it. An individual's consumption pattern can be modified by choosing to consume energy from local sources rather than purchase energy from the SMG. As a result, SMG demand curves have a significant impact on energy efficiency. As such, the suggested DSM considers PAR demand as a technical indicator. A comparison of the per-day average demand of all devices to the peak consumption of all appliances is made using the index. Eq. (29) calculates the SMG average demand during a day.

(29) $$Ave_{D}=\frac{\sum_{t=1}^{T}P_{SH}\left(t\right)}{T}$$

In which, $P_{SH}\left(t\right)$ shows the overall bought energy from MG during time t, and T shows the number of time intervals.

Eq. (30) calculates the SG peak demand during a day.

(30) $$P_{D}=\max\sum_{t=1}^{T}P_{SH}\left(t\right)$$

Finally, Eq. (31) calculates the index of the PAR demand of SMG.

(31) $$OF_{2}=P_{D}/Ave_{D}$$

Based on Eq. (31), whenever the index is close to one, the suggested operation time of equipment works optimally.

## 4 BBO algorithm

BBO algorithm can be defined as a population-based evolutionary algorithm using migration and mutation concepts to determine the characteristics of the solutions in a probabilistic manner [41]. This algorithm differs from other algorithms in several ways which are shortly discussed below [42]. As in PSO, BBO does not eliminate solutions after every generation, as opposed to differential evolution (DE) and GA. BBO optimizes solutions in the migration method, whereas evolutionary algorithms lose their quality as a result of crossover operations. PSO solutions, however, tended to stick together more often than BBO solutions due to the mutation procedure. Among the special features of the BBO algorithm is the ability to improve poor solutions by incorporating characteristics from better solutions. As a result of this characteristic, constraint satisfaction occurs more easily compared to the bacterial foraging algorithm [42].

BBO's mathematical model illustrates how species migrate and emigrate between various habitats. Habitat suitability index (HSI) is a measure of whether a habitat is suitable for a species [43]. A further parameter that characterizes habitability is the suitability index variable (SIV) [41]. Habitats with high HSI are home to the majority of species. Because of their large populations, habitats suffer from low immigration and high emigration rates. In contrast, habitats with low HSI are home to fewer species. Additionally, habitats with low HSI tend to have high immigration and low emigration ratios because of their small populations. Immigration of new species could enhance habitats with low HSI. A low HSI may also lead to the extinction of the species.

**Fig. 2:**
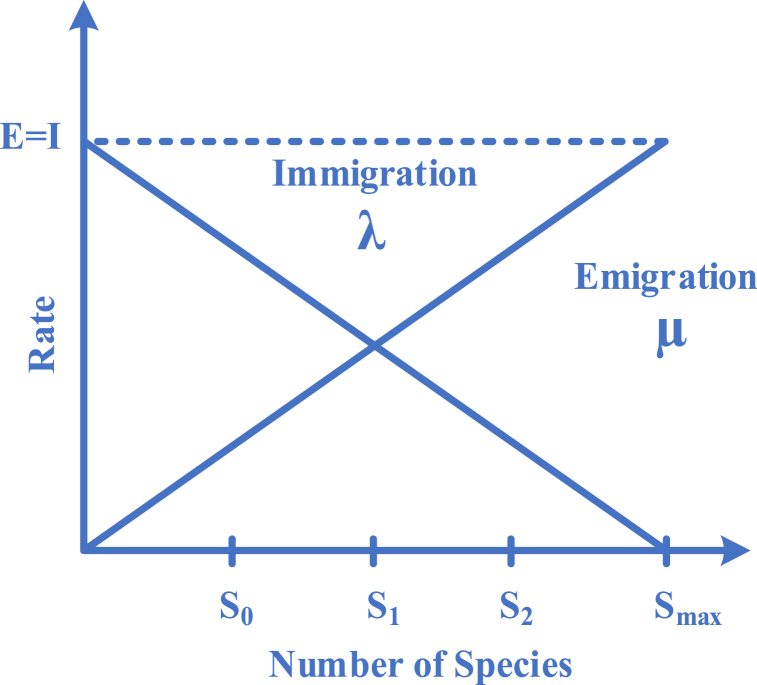
Emigration, and immigration rate model.

HSI represents the fitness of each solution. Habitats define probable solutions of the OP. Fig. 2 shows a model of species abundance, where λ is the immigration rate, μ is the emigration rate, and S shows species number. The maximum possible immigration rate is defined by I and the maximum possible emigration rate is defined by E. In order to simplify the analysis, emigration, and immigration habitats are assumed to be linear functions of habitats HSI and E and I do not differ. A habitat's species density is also a factor in determining these rates.

Eq. (32) and Eq. (33) are used to calculate the immigration and emigration rates for every solution $H_{i}$, in which, $k_{i}$ shows the number of species. n shows the maximum number of species.

(32) $$\mu =E\left(\frac{k_{i}}{n}\right)$$

(33) $$\lambda =I\left(1-\frac{k_{i}}{n}\right)$$

The probabilistic scheme illustrates the mathematical model for the emigration process and immigration process. $P_{s}$ shows the probability in which *S* species would be in the habitat during time t. The probability between t to t+Δt is shown in Eq. (34), in which, $\mu_{s}$ is the emigration rate, $\lambda_{s}$ is the immigration rate, and S species exist in the habitat. Eq. (35) has been defined as the restriction of Eq. (34) in a way that Δt reaches 0.

(34) $$P_{s}\left(t+\Delta t\right)=P_{s}\left(t\right)\left(1-\lambda_{s}\Delta t-\mu_{s}\Delta t\right)+P_{s-1}\lambda_{s-1}\Delta t+P_{s+1}\mu_{s+1}\Delta t$$

(35) $$P_{s}=\left\{ \begin{array}{c} -\left(\lambda_{s}+\mu_{s}\right)P_{s}+\mu_{s+1}P_{s+1}\;S=0\\ -\left(\lambda_{s}+\mu_{s}\right)P_{s}+\lambda_{s-1}P_{s-1}+\mu_{s+1}P_{s+1}\;1\leq S\leq S_{\max}\\ -\left(\lambda_{s}+\mu_{s}\right)P_{s}+\lambda_{s-1}P_{s-1}\;S=S_{\max}\{ \end{array}\right.$$

**Fig. 3:**
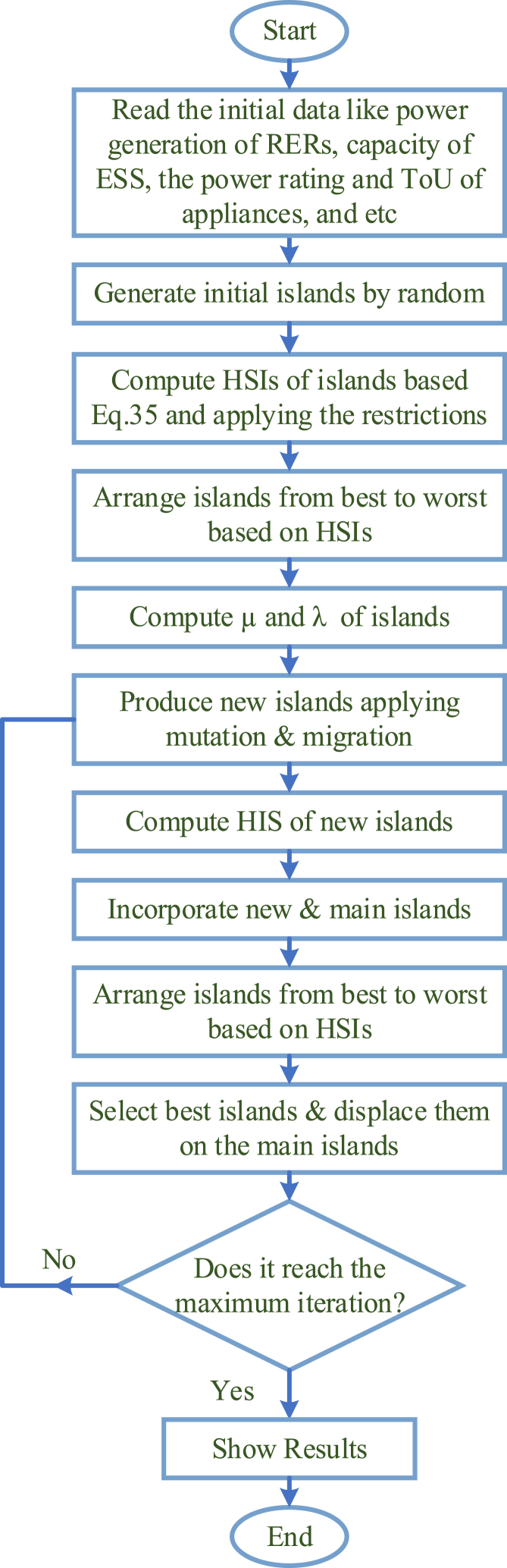
Flowchart diagram of the BBO algorithm.

In [44], a step-by-step guide is provided for executing the BBO algorithm. Fig. 3 illustrates the proposed algorithm's flowchart.

Step 1: Input amounts (including the number of variables, the maximum number of iterations, and population size) are adjusted. Here, N represents the population number, 260 in this case. $m_{i}$ describes the number of species in habitat i, and 1000 is set to the maximum number of iterations. The parameters of the BBO should be initialized such as habitat modification probability $P_{\mathrm{mod}}$, immigration and maximum emigration ratios, and mutation probability $P_{mutation}$. A keep-rate of 0.2 means that 20 percent of the best habitats remain unchanged while 80 percent are modified. Lastly, the algorithm's hyperparameters should be α=0.9, and $\delta =0.02*\left(X_{\max}-X_{\min}\right)$.

Step 2: The primary population is generated randomly. In addition, the initial population must satisfy the restrictions. Maximums and minimums of variables were used to compute $X_{\max}$ and $X_{\min}$. Eq. (36) is then used to calculate the initial population. Where rand shows a randomly selected vector between 0 and 1.

(36) $${\bar{X}}_{pop}=rand*\left(X_{\max}-X_{\min}\right)+X_{\min}$$

The initial population is calculated by repeating the process for all parameters, for all population numbers.

Step 3: By applying Eq. (37), any restrictions are released:

(37) $$G\left(X\right)=\left[f\left(\bar{X}\right)\right]+L_{1}\left(\sum_{1}^{N_{\text{eq}}}{\left(h_{\text{eq}}\left(\bar{X}\right)\right)}^{2}\right)+L_{2}\left(\sum_{1}^{N_{\text{ineq}}}{\left(\mathrm{Max}\left[0,‐g_{ineg}\left(\bar{X}\right)\right]\right)}^{2}\right)$$

Here, $f\left(\bar{X}\right)$ shows the of the problem, $g_{ineg}\left(\bar{X}\right)$ is the inequality limitation and $h_{eq}\left(\bar{X}\right)$ is the equality restriction. Penalty values $L_{1}$ and $L_{2}$ affect the model's efficiency. The method could get stuck in the local minima with too high penalty values. A penalty value that is too low can also prevent the algorithm from determining the feasible and optimal solution. The penalty variables were determined by extracting an adaptive penalty function from Ref. [45].

For simplicity, $\sum_{1}^{N_{eq}}{\left(h_{eq}\left(\bar{X}\right)\right)}^{2}$ and $\sum_{1}^{N_{ineq}}{\left(\mathrm{Max}\left[0,g_{ineq}\left(\bar{X}\right)\right]\right)}^{2}$ is described as B and $L_{1}=L_{2}=i\sqrt{i}\lambda \beta^{\delta }$. Where i shows the iteration number. δ is the power of the penalty value. λ shows a multi-step assignment value. When B≤1 so δ=1, if not, δ=2. Moreover, when B≤0.001, so λ=1, otherwise B≤0.01, so λ=10, otherwise B≤0.1, so λ=30, if not, λ=100.

Step 4: The OF should be calculated based on the immigration rate and emigration rate. A population is ordered according to the values of the OF. Next, the sorted population (${\bar{X}}_{pop-sorted}$) and best answer ($X_{best}$) are determined.

Step 5: The probabilistic migration is performed on SIVs with populations 80 percent lower than that of the ${\bar{X}}_{pop-sorted}$. The roulette wheel selection should be based on the emigration rate when selecting emigrating islands. The first step is to calculate the emigration rate and immigration rate for all habitat sets. Then, once migration has been completed, new habitats and SIVs will be selected.

Step 6: Probabilistically mutation should be performed based on $P_{mutation}$ for all habitats.

Step 7: The OF for all mutated habitats should be calculated.

Step 8: $X_{pop}$ should be updated and Step 4 should be repeated as many times as necessary until the maximum number of iterations has been reached.

## 5 Simulation results

### 5.1 Optimal scheduling of different schemes

The following part presents the simulation outcomes. An energy consumption simulation of home devices is performed every hour of the day. There are 48 time slots in a day. The problem is solved using the BBO algorithm in multi-objective mode with the Pareto front of the optimal solutions which considers electricity cost and PAR in different cases as follows:

Case-1: Considering the proposed SHEM in single objective mode (consider Electricity price),

Case-2: Considering the proposed SHEM with and without the different sizes of RERs,

Case-3: Considering the proposed SHEM under different charging/discharging rates of ESS,

Case-4: Considering the proposed SHEM under different selling price costs of RERs,

Case-5: Considering the proposed SHEM with Costs of RERs.

The program runs on Intel(R) Core(TM) i7-4600U CPU @ 2.10 GHz and 12 GB RAM with Windows 10 pro. The time slots used in this paper are specified by the indices shown in Table 2, for example, index 1 represents the time interval 00:00 to 00:30. The detail of the proposed system including DAP signal, hourly PV, and WT generation is described in Table 3. Table 4 describes the loads’ details including power rating and operational time of shiftable and non-shiftable loads. The ESS parameters are described in Table 5.

**Table 2:** Indicators representing each time slot.

Time Slot	Index	Time Slot	Index	Time Slot	Index	Time Slot	Index
00:00–00:30	1	06:00–06:30	13	12:00–12:30	25	18:00–18:30	37
00:30–01:00	2	06:30–07:00	14	12:30–13:00	26	18:30–19:00	38
01:00–01:30	3	07:00–07:30	15	13:00–13:30	27	19:00–19:30	39
01:30–02:00	4	07:30–08:00	16	13:30–14:00	28	19:30–20:00	40
02:00–02:30	5	08:00–08:30	17	14:00–14:30	29	20:00–20:30	41
02:30–03:00	6	08:30–09:00	18	14:30–15:00	30	20:30–21:00	42
03:00–03:30	7	09:00–09:30	19	15:00–15:30	31	21:00–21:30	43
03:30–04:00	8	09:30–10:00	20	15:30–16:00	32	21:30–22:00	44
04:00–04:30	9	10:00–10:30	21	16:00–16:30	33	22:00–22:30	45
04:30–05:00	10	10:30–11:00	22	16:30–17:00	34	22:30–23:00	46
05:00–05:30	11	11:00–11:30	23	17:00–17:30	35	23:00–23:30	47
05:30–06:00	12	11:30–12:00	24	17:30–18:00	36	23:30–24:00	48

**Table 3:** Hourly details of the proposed system.

Time of the day (30 min)	DAP signal (U.S. cents/kWh)	PV Generation (kW)	WT Generation (kW)	Time of the day (30 min)	DAP signal (U.S. cents/kWh)	PV Generation (kW)	WT Generation (kW)
1	10	0	0.2455	25	16.5	0.97	0.5385
2	10	0	0.2455	26	16.5	0.97	0.5385
3	10	0	0.2455	27	16.2	0.97	0.326
4	10	0	0.2455	28	16.2	0.97	0.326
5	8.5	0	0.2455	29	14	0.97	0.2455
6	8.5	0	0.2455	30	14	0.97	0.2455
7	9	0	0.2455	31	9.2	0.97	0.1795
8	9	0	0.2455	32	9.2	0.97	0.1795
9	12.2	0	0.2455	33	8.5	0.81	0.2455
10	12.2	0	0.2455	34	8.5	0.81	0.2455
11	9.2	0	0.126	35	8.8	0.65	0.2455
12	9.2	0	0.126	36	8.8	0.65	0.2455
13	12.3	0	0.2455	37	9.7	0.41	0.179
14	12.3	0	0.2455	38	9.7	0.41	0.179
15	24.7	0.33	0.1795	39	8	0.12	0.2455
16	24.7	0.33	0.1795	40	8	0.12	0.2455
17	27	0.73	0.2455	41	8.2	0	0.179
18	27	0.73	0.2455	42	8.2	0	0.179
19	27.5	0.81	0.425	43	8	0	0.179
20	27.5	0.81	0.425	44	8	0	0.179
21	17	0.81	1.2065	45	8.2	0	0.126
22	17	0.81	1.2065	46	8.2	0	0.126
23	16.5	0.97	1.4315	47	8.2	0	0.0845
24	16.5	0.97	1.4315	48	8.2	0	0.0845

**Table 4:** Hourly details of the loads.

Non-Shiftable Loads				Continuous Shiftable Loads				Interruptible Shiftable Loads			
Load	Power Rating (kW)	Daily Usage (hr)	Start time	Load	Power Rating (kW)	Daily Usage (hr)	Time slot	Load	Power Rating (kW)	Daily Usage (hr)	Time slot
Refrigerator	0.9	20	2:00	Bread Machine	0.6	4	6:00∼10:00	Main Air Conditioner	2.5	8	1:00∼7:00
Personal Computers	0.3	18	7:00	Spin Dryer	2.5	4	9:00∼22:00	Living room Air Conditioner	1.5	6	9:00∼22:00
Microwave Oven	1.7	7	15:00	Cooker Oven	3	4	10:00∼20:00	Workroom Air Conditioner	1.5	4	9:00∼20:00
Security Cameras	0.1	24	00:00	Coffee Machine	0.3	2	6:00∼10:00	Water Heater	2	16	7:00∼22:00
Television	0.2	8	16:00	Washing Machine	0.85	4	6:00∼24:00	Laptop	0.1	4	8:00∼23:00
Lights	0.2	6	18:00	Dish Washer	1.45	6	10:00∼22:00	Disinfection cabinet	0.6	6	6:00∼24:00
				Music center	0.15	4	17:00∼23:00	EV	3.3	6	1:00∼7:00
				Bath heater	1	2	20∼23	Bedroom Air Conditioner-1	1.5	6	1:00∼8:00
				Electric kettle	0.5	4	1:00∼19:00	Bedroom Air Conditioner-2	1.5	6	1:00∼10:00
				Juicer	0.5	2	6:00∼23:00

**Table 5:** Details of ESS.

Parameter	$SoE_{initial}$ (kW)	$SoE_{\min}$ (kW)	$SoE_{\max}$ (kW)	$R_{Bat}^{ch}/R_{Bat}^{dis}$ (kW/Time Slot)
Value	0.5	0.5	3	0.3

### 5.2 Case-1: Considering the proposed SHEM in single objective mode

**Fig. 4:**
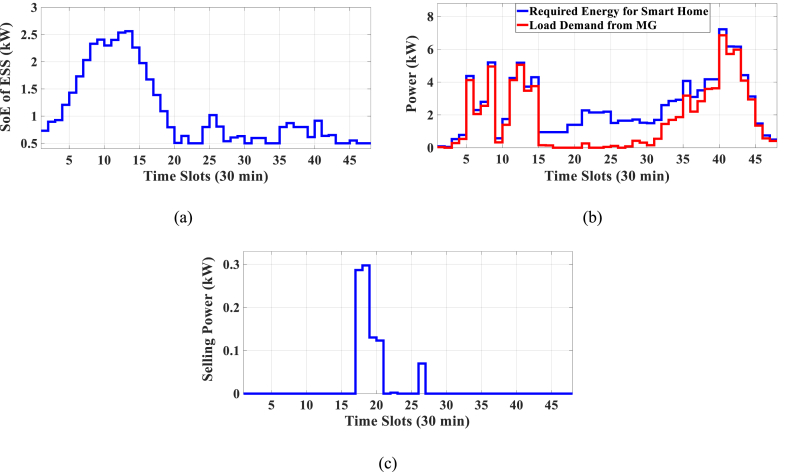
Single objective mode considering $OF_{1}$: a) SoE of ESS, b) hourly load demand from MG, c) hourly selling energy to the MG.

In this case study, the PAR or Electricity cost is optimized separately. The selling electricity tariff ratio is ρ=0.9. The results are shown in Table 6. As can be seen, the electricity cost is 747.851 U.S. cents, and it is reduced by 21 % compared to unscheduled mode. Also, for optimized PAR in single objective mode, PAR 1.629 is obtained which is a reduction of 46 % as compared to unscheduled mode. As shown in Table 6, by optimizing one of the objective functions, the other is increased, so both objective functions should be optimized simultaneously in multi-objective mode. The results of SoE, load demand, and selling power to MG for single objective mode considering electricity cost are shown in Fig. 4. Fig. 4a shows that the ESS is charged in low-price time slots and discharged in high-price time slots to reduce the entire cost of the system. The required energy for SH and energy demand from MG are shown in Fig. 4b. As can be seen in Fig. 4c, the surplus energy is sold to MG in the high-price time slot. Shiftable load scheduling for the single objective mode that considers the electricity cost is illustrated in Table 7. In this case study, it was shown that considering only the cost objective function increases the PAR value, which is a technical factor in operation. Therefore, it is important and necessary to consider both objective functions, optimize them together, and solve the problem as a multi-objective problem.

**Table 6:** Entire Energy cost of Case-1.

Method	Cost (U.S. cents)	Cost Reduction (%)	PAR	PAR Reduction (%)
Unscheduled	947.013	–	3.025	–
BBO Considering $OF_{1}$	747.851	21	3.932	–
BBO Considering $OF_{2}$	1063.185	–	1.629	46

**Table 7:** Shiftable Loads scheduling for single objective mode considering $OF_{1}$.

Shiftable Loads	Used time slots	Shiftable Loads	Used time slots	Shiftable Loads	Used time slots	Shiftable Loads	Used time slots
Bread Machine	15∼18	Dish Washer	37∼42	Main Air Conditioner	5, 7, 8, 10∼14	Disinfection cabinet	43∼48
Spin Dryer	40∼43	Music center	34∼37	Living room Air Conditioner	25, 40∼44	EV	5, 8, 11∼14
Cooker Oven	21∼24	Bath heater	40, 41	Workroom Air Conditioner	35, 38∼40	Bedroom Air Conditioner-1	5∼8, 12, 14,
Coffee Machine	12, 13	Electric kettle	33∼36	Water Heater	26∼28, 32∼44	Bedroom Air Conditioner-2	6, 8, 11, 12, 19, 20
Washing Machine	42∼45	Juicer	44, 45	Laptop	42, 43, 45, 46		

### 5.3 Case-2: Considering the proposed SHEM with different sizes of RERs

In this case, the HEMS is considered without and with RERs in 3 states such as without RERs, with only a PV system, and considered both PV and WT together. The selling electricity tariff ratio is ρ=0.9 for this case study.

#### 5.3.1 Without RER

**Fig. 5:**
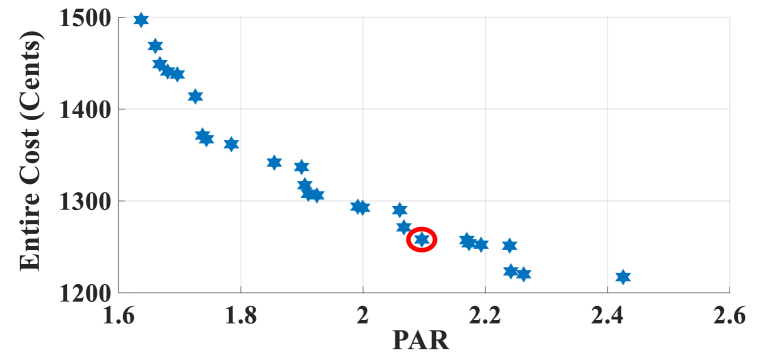
Multi-objective Pareto optimal solution set for case-2 without RERs.

**Fig. 6:**
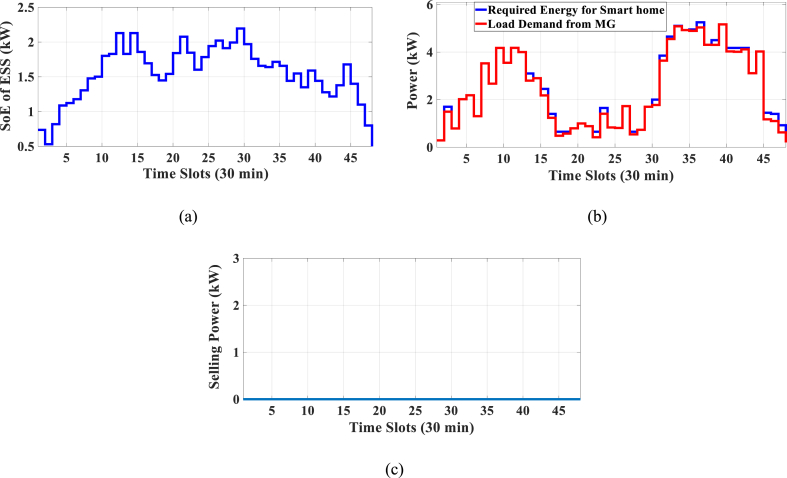
MOEMS for case-2 without RERs (PV and WT) for marked solution in Fig. 5 with electricity cost equal to 1258.02 U.S. cents and PAR equal to 2.096: a) SoE of ESS, b) Hourly load demand from MG, c) Hourly selling energy to the MG.

Here, the HEMS is considered sans RERs, and only ESS participates in energy management. The Pareto Front of the optimal solutions for this subsection is shown in Fig. 5. The results of SoE, load demand, and selling power to MG for Multi-objective energy management system (MOEMS) for case-2 without RERs (PV and WT) for marked solution with electricity cost equal to 1258.02 U.S. cents and PAR equal to 2.096 are shown in Fig. 6. Fig. 6a is shown that the ESS is charged in low price time slots and discharged in high price time slots to reduce the entire cost of the system. The required energy for SH and energy demand from MG are shown in Fig. 6b. As can be seen in Fig. 6b, by participating the ESS in HEMS, the energy demand is lower than the required energy for SH. As shown in Fig. 6c, the selling energy is zero because there are no RERs in this scenario. Shiftable load scheduling for the marked solution is illustrated in Table 8, by specifying the used time slots for each appliance.

**Table 8:** Shiftable Loads scheduling for case-2 without RERs (PV and WT) for marked solution in Fig. 5 with electricity cost equal to 1258.02 U.S. cents and PAR equal to 2.096.

Shiftable Loads	Used time slots	Shiftable Loads	Used time slots	Shiftable Loads	Used time slots	Shiftable Loads	Used time slots
Bread Machine	12∼15	Dish Washer	39∼44	Main Air Conditioner	4, 7∼13	Disinfection Cabinet	32, 36, 38, 44, 47, 48
Spin Dryer	36∼39	Music center	43∼46	Living room Air Conditioner	36, 40∼44	EV	2, 5, 7, 9, 11, 12
Cooker Oven	32∼35	Bath heater	45, 46	Workroom Air Conditioner	31, 33–35,	Bedroom Air Conditioner-1	8∼10, 14∼16
Coffee Machine	13, 14	Electric kettle	30∼33	Water Heater	23, 26, 31∼44	Bedroom Air Conditioner-2	6, 10, 11, 13∼15
Washing Machine	44∼47	Juicer	30, 31	Laptop	19, 20, 44, 45		

#### 5.3.2 Without WT

**Fig. 7:**
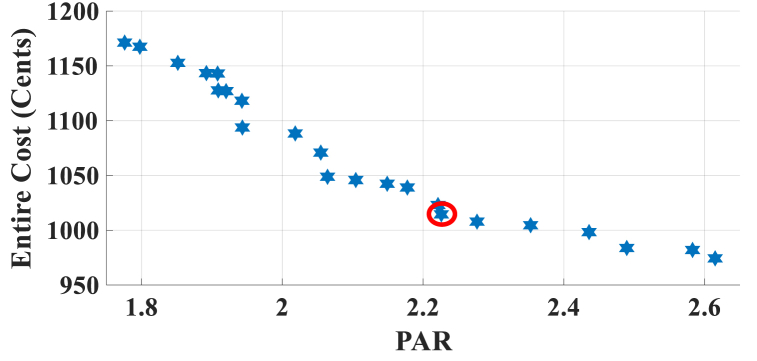
Multi-objective Pareto optimal solution set for case-2 with PV and without WT.

**Fig. 8:**
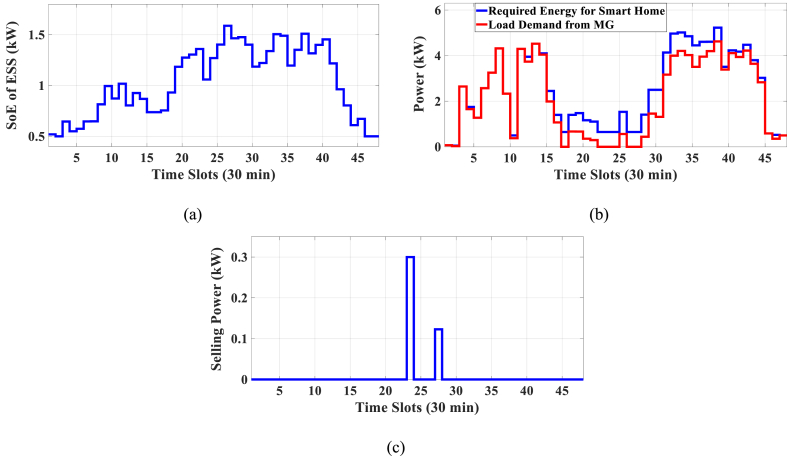
MOEMS for case-2 with PV and without WT for marked solution in Fig. 7 with electricity cost equal to 1014.525 U.S. cents and PAR equal to 2.226: a) SoE of ESS, b) Hourly load demand from MG, c) Hourly selling energy to the MG.

Here, the HEMS is considered with PV, ESS, and sans WT. The Pareto Front of the optimal solutions for this subsection is shown in Fig. 7. The results of SoE, load demand, and selling power to MG of MOEMS for marked solution in Fig. 7 with electricity cost equal to 1014.525 U.S. cents and PAR equal to 2.226 are shown in Fig. 8.

Fig. 8a depicts the SoE of ESS, as can be seen, the ESS is charged by PV, and MG in low-price time slots and discharged to fed loads and selling energy in high-price time slots to reduce the entire cost of the system. The required energy for SH and energy demand from MG is shown in Fig. 8b. As can be seen in Fig. 8b, by participating ESS and PV in HEMS, the energy demand is lower than the required energy for SH especially when the PV system produces energy. As shown in Fig. 8c, the energy is sold to the MG in high-price time slots. Shiftable load scheduling for the marked solution is illustrated in Table 9.

**Table 9:** Shiftable Loads scheduling for case-2 with PV and without WT for marked solution in Fig. 7 with electricity cost equal to 1014.525 U.S. cents and PAR equal to 2.226.

Shiftable Loads	Used time slots	Shiftable Loads	Used time slots	Shiftable Loads	Used time slots	Shiftable Loads	Used time slots
Bread Machine	12∼15	Dish Washer	38∼43	Main Air Conditioner	3, 4, 6–8, 11–13,	Disinfection Cabinet	13, 42–44, 47, 48
Spin Dryer	31∼34	Music center	43∼46	Living room Air Conditioner	25, 40∼44	EV	8, 9, 11∼14
Cooker Oven	35∼38	Bath heater	43, 44	Workroom Air Conditioner	28, 32∼34	Bedroom Air Conditioner-1	5, 7, 8, 11, 14, 15
Coffee Machine	18, 19	Electric kettle	35∼38	Water Heater	29∼44	Bedroom Air Conditioner-2	3, 6, 7, 14∼16
Washing Machine	18∼21	Juicer	12, 13	Laptop	38, 43, 45, 46		

#### 5.3.3 With RERs

**Fig. 9:**
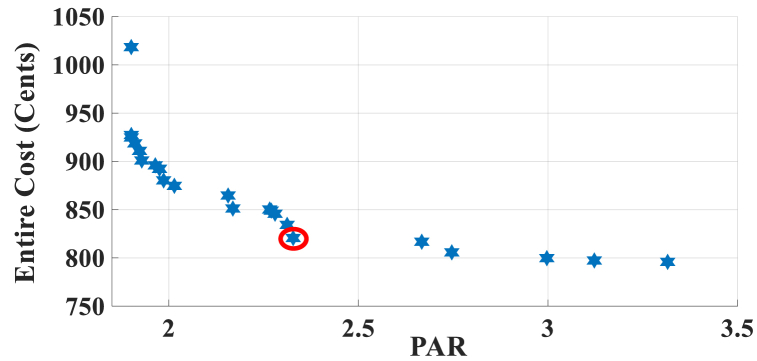
Multi-objective Pareto optimal solution set for case-2 with RERs (PV and WT).

**Fig. 10:**
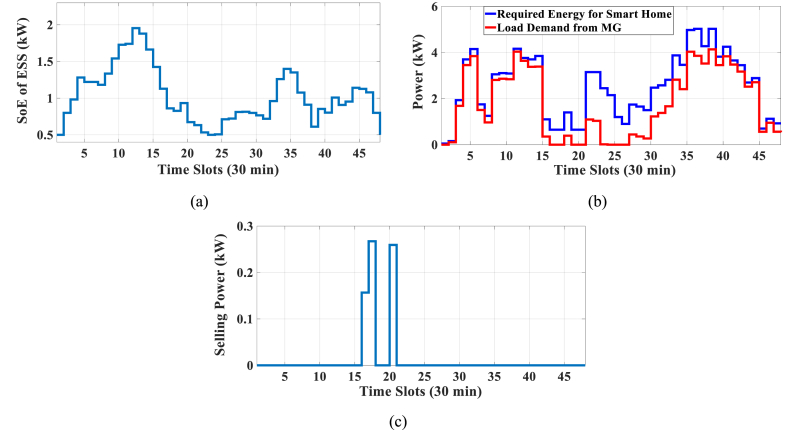
MOEMS for case-2 with RERs (PV and WT) for marked solution in Fig. 9 with electricity cost equal to 820.74 U.S. cents and PAR equal to 2.32: a) SoE of ESS, b) Hourly load demand from MG, c) Hourly selling energy to the MG.

Here, the HEMS is considered with PV, WT, and ESS. The Pareto Front of the optimal solutions for this scenario is shown in Fig. 9. The results of SoE, load demand, and selling power to MG of MOEMS for marked solution in Fig. 9 with electricity cost equal to 820.74 U.S. cents and PAR equal to 2.32 are shown in Fig. 10. Fig. 10a is shown the SoE of ESS, as can be seen, the ESS is charged by RERs, and MG in low price time slots and discharged to fed loads and selling energy in high price time slots to reduce the entire cost of the system. The required energy for SH and energy demand from MG are shown in Fig. 10b, and can be seen, that the energy demand is lower than the required energy for SH especially in high-price time slots. As shown in Fig. 10c, the energy is sold to the MG in high-price time slots. Shiftable load scheduling for the marked solution is illustrated in Table 10.

**Table 10:** Shiftable Loads scheduling for case-2 with RERs (PV and WT) for marked solution in Fig. 9 with electricity cost equal to 820.74 U.S. cents and PAR equal to 2.32.

Shiftable Loads	Used time slots	Shiftable Loads	Used time slots	Shiftable Loads	Used time slots	Shiftable Loads	Used time slots
Bread Machine	12∼15	Dish Washer	30∼35	Main Air Conditioner	3∼6, 11∼14	Disinfection Cabinet	23, 25, 45∼48
Spin Dryer	35∼38	Music center	36∼39	Living room Air Conditioner	36, 40∼44	EV	4, 5, 8–11,
Cooker Oven	21∼24	Bath heater	43, 44	Workroom Air Conditioner	18, 38∼40	Bedroom Air Conditioner-1	5, 8, 9, 12∼14
Coffee Machine	14, 15	Electric kettle	30∼33	Water Heater	21, 22, 27, 28, 33∼34	Bedroom Air Conditioner-2	7, 10∼15
Washing Machine	45∼48	Juicer	25, 26	Laptop	35, 39, 40, 44		

Figs, 8 and 10 show that by participating the RERs, the entire cost is reduced, and also by increasing the produced energy of RERs, the selling energy is increased.

In this case study, it was shown that without considering RERs, the amount of energy purchased from the upstream grid increases, which also minimizes the selling energy (According to international scientific consensus, to prevent worsening climate damage, net human-caused carbon dioxide ($CO_{2}$) emissions need to be reduced by about 45 % from 2010 to 2030, and also reach zero by 2050 [40]. Hence, in this paper in order to maximize the use of RERs, we assumed, the energy cost of WT, and PV are zero to use the maximum energy of RERs).

Without considering RERs, the cost is 1258.02 and the PAR is 2.096 while considering the PV, the cost is 1014.525 U.S. cents and the PAR is 2.226, and also considering the PV and WT simultaneously, the cost is 820.74 U.S. cents and the PAR is 2.32 (the results are related to the selected solution from the set of solutions). The results in this case study show that by using the PV system, the cost is reduced by 20 percent, but the PAR is increased 6 %. Also, considering the PV and WT simultaneously, the cost is reduced by 34.7 %, but the PAR is increased by 10.6 %.

### 5.4 Case-3: Considering the proposed SHEM under different charging rates of ESS

In this case study, the HEMS is considered under different kinds of charging rates such as 0.3, 0.6, and 0.9 (kW/Time Slot). In this case, the system includes the PV, WT, and ESS. The results of SHEM with ${Ch}_{rate}=0.3$ (kW/Time Slot) are performed in the prior subsection (Case-2, subsection 3). The results of the SHEM system with ${Ch}_{rate}=0.6$ and 0.9 (kW/Time Slot) are investigated in the following. The selling electricity tariff ratio is ρ=0.9 for this case study.

#### 5.4.1 $Ch_{\mathrm{rate}}=0.6$ (kW/Time slot)

**Fig. 11:**
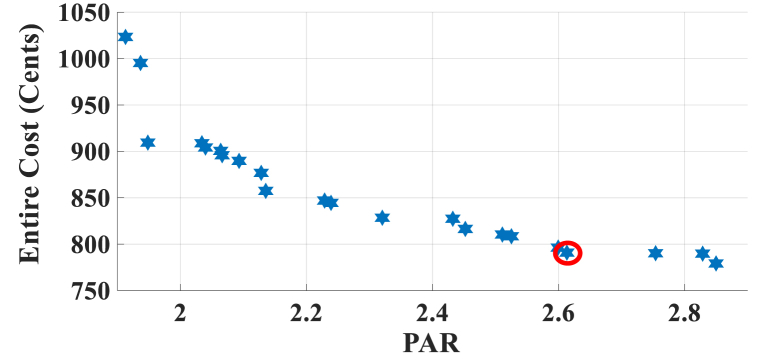
Multi-objective Pareto optimal solution set for case-3 for $Ch_{rate}=0.6$.

**Fig. 12:**
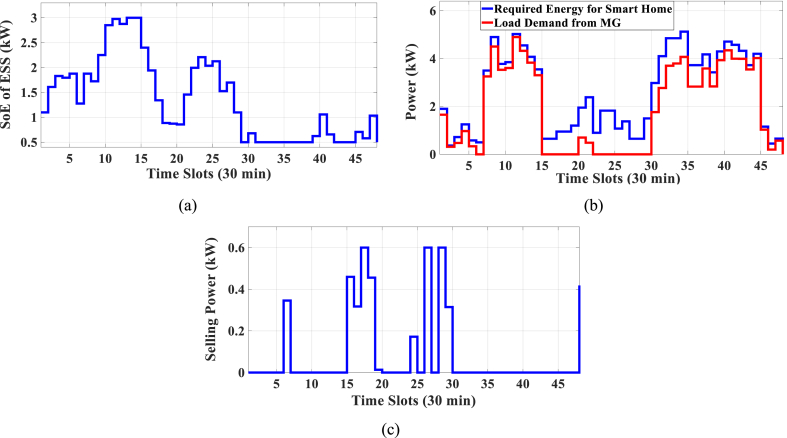
MOEMS for case-3 for $Ch_{rate}=0.6$ for marked solution in Fig. 11 with electricity cost equal to 790.873 U.S. cents and PAR equal to 2.613: a) SoE of ESS, b) Hourly load demand from MG, c) Hourly selling energy to the MG.

In this subsection, the results of HEMS with ${Ch}_{rate}=0.6$ are depicted in Fig. 11, Fig. 12. Fig. 11 displays the Pareto Front of the optimal solutions for this scenario. The results of SoE, load demand, and selling power to MG of MOEMS for marked solution in Fig. 11 with electricity cost equal to 790.873 U.S. cents and PAR equal to 2.613 are shown in Fig. 12.

Fig. 12a shows the SoE of ESS, as can be seen, the ESS is charged by RERs, and MG in low-price time slots and discharged to fed loads and selling energy in high-price time slots to reduce the entire cost of the system. The required energy for SH and energy demand from MG are shown in Fig. 12b, and can be seen, that the energy demand is lower than the required energy for SH especially in high-price time slots. As shown in Fig. 12c, the energy is sold to the MG in high-price time slots. Shiftable load scheduling for the marked solution is illustrated in Table 11.

**Table 11:** Shiftable Loads scheduling for case-3 for $Ch_{rate}=0.6$ for marked solution in Fig. 11 with electricity cost equal to 790.873 U.S. cents and PAR equal to 2.613.

Shiftable Loads	Used time slots	Shiftable Loads	Used time slots	Shiftable Loads	Used time slots	Shiftable Loads	Used time slots
Bread Machine	17∼20	Dish Washer	34∼39	Main Air Conditioner	1, 8∼14	Disinfection Cabinet	26, 30, 34–36, 41
Spin Dryer	41∼44	Music center	40∼43	Living room Air Conditioner	24, 32, 33, 40, 43, 44	EV	7, 8, 11∼14
Cooker Oven	31∼34	Bath heater	44, 45	Workroom Air Conditioner	23, 37, 39, 40	Bedroom Air Conditioner-1	4, 8–12,
Coffee Machine	12, 13	Electric kettle	19∼22	Water Heater	21, 30∼44	Bedroom Air Conditioner-2	7∼11, 20
Washing Machine	23∼26	Juicer	12, 13	Laptop	42, 44∼46		

#### 5.4.2 $Ch_{\mathrm{rate}}=0.9$ (kW/Time slot)

**Fig. 13:**
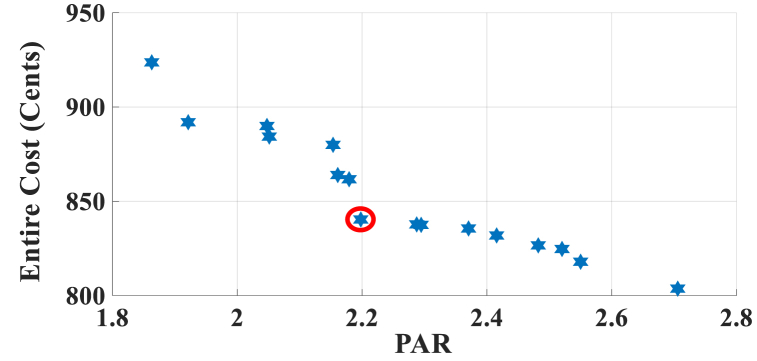
Multi-objective Pareto optimal solution set for case-3 for $Ch_{rate}=0.9$.

**Fig. 14:**
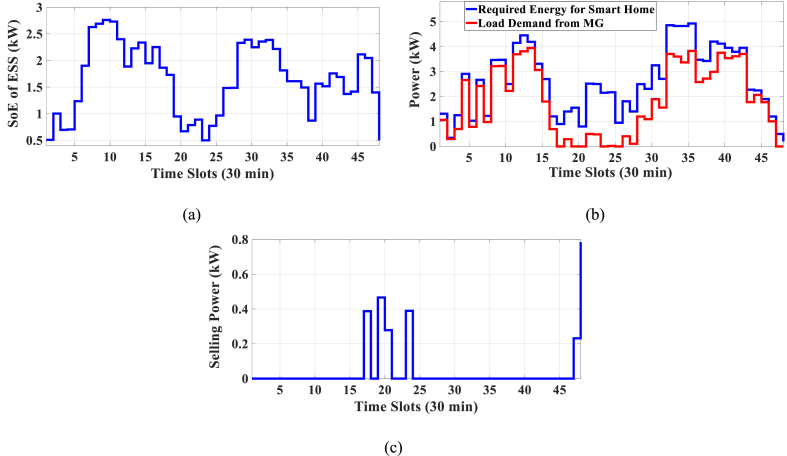
MOEMS for case-3 for $Ch_{rate}=0.9$ for marked solution in Fig. 13 with electricity cost equal to 840.358 U.S. cents and PAR equal to 2.198: a) SoE of ESS, b) Hourly load demand from MG, c) Hourly selling energy to the MG.

In this subsection, the results of HEMS with ${Ch}_{rate}=0.9$ are depicted in Fig. 13, Fig. 14. Fig. 13 displays the Pareto Front of the optimal solutions for this scenario. The results of SoE, load demand, and selling power to MG for MOEMS for marked solution in Fig. 13 with electricity cost equal to 840.358 U.S. cents and PAR equal to 2.198 are shown in Fig. 14. Fig. 14a is shown the SoE of ESS, as can be seen, the ESS is charged by RERs, and MG in low price time slots and discharged to fed loads and selling energy in high price time slots to reduce the entire cost of the system. The required energy for SH and energy demand from MG are shown in Fig. 14b. As shown in Fig. 14c, the energy is sold to MG in high-price time slots. Shiftable load scheduling for the marked solution is illustrated in Table 12.

**Table 12:** Shiftable Loads scheduling for case-3 for $Ch_{rate}=0.9$ for marked solution in Fig. 13 with electricity cost equal to 840.358 U.S. cents and PAR equal to 2.198.

Shiftable Loads	Used time slots	Shiftable Loads	Used time slots	Shiftable Loads	Used time slots	Shiftable Loads	Used time slots
Bread Machine	12∼15	Dish Washer	32∼37	Main Air Conditioner	1, 8∼14	Disinfection Cabinet	25, 39, 41, 45∼47
Spin Dryer	21∼24	Music center	40∼43	Living room Air Conditioner	29, 30, 38, 42∼44	EV	4, 8, 9, 11∼13
Cooker Oven	32∼35	Bath heater	45, 46	Workroom Air Conditioner	26, 27, 38, 40	Bedroom Air Conditioner-1	4, 5, 10–12, 15
Coffee Machine	19, 20	Electric kettle	14∼17	Water Heater	28, 30∼44	Bedroom Air Conditioner-2	3, 6, 14, 15, 18, 19
Washing Machine	39∼42	Juicer	21, 22	Laptop	36, 41, 43, 44		

The results in Case-4 show that by increasing the charging/discharging rate, the ESS participates more in HEMS, and the selling energy is increased. For $Ch_{rate}=0.3$, the SoE of ESS is not fully charged, and the entire capacity of ESS is not used, but by increasing the charging rate, the entire capacity of ESS is used.

In this case study, the charging/discharging rate of ESS is considered. When the ESS charging/discharging rate is 0.3 (kW/Time slot) (case study-2, subsection 3), the electricity cost is 820.74 U.S. cents and PAR is 2.32. For the ESS charging/discharging rate with 0.6 and 0.9 (kW/Time slot), the electricity costs are 790.873 U.S. cents and 840.358 U.S. cents, and PAR are 2.613, and 2.198, respectively. As can be seen, by increasing the charging/discharging rate from 0.3 to 0.6, the electricity cost is reduced by 3.6 %, and the PAR is increased by 12.6 %. Also, by increasing the charging/discharging rate from 0.3 to 0.9, the electricity cost is increased by 2.4 %, and the PAR is reduced by 5.3 %. By increasing the charging/discharging rate from 0.6 to 0.9, the electricity cost is increased by 6.3 %, and the PAR is reduced by 18.9 %.

### 5.5 Case-4: Considering the proposed SHEM under different selling electricity tariff ratio

In this case study, the HEMS is considered under different selling electricity tariff ratios such as ρ=0.8, 0.9, and 1. The considered system in this case includes PV, WT, and ESS with $Ch_{rate}=0.6$. The results of the SHEM system with ρ=0.9 are performed in the prior subsection (Case-3, subsection 1). The results of the SHEM system with ρ=1 and ρ=0.8 are investigated in the following.

#### 5.5.1 ρ=1

**Fig. 15:**
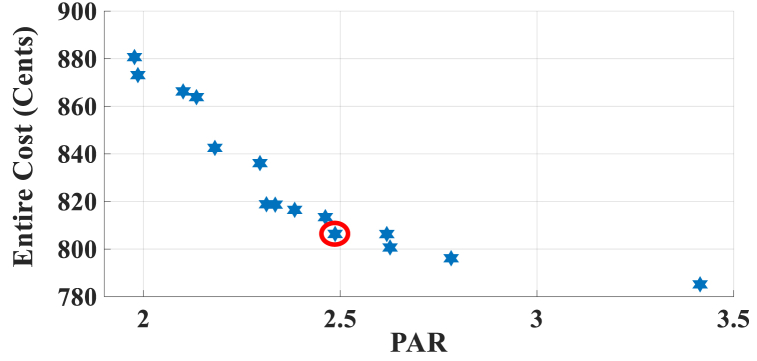
Multi-objective Pareto optimal solution set for case-4 with ρ=1.

**Fig. 16:**
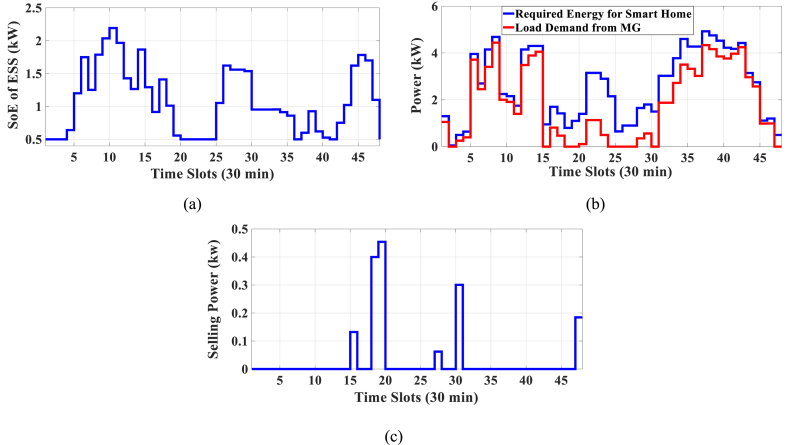
MOEMS for case-4 with ρ=1 for marked solution in Fig. 15 with electricity cost equal to 806.363 U.S. cents and PAR equal to 2.487: a) SoE of ESS, b) Hourly load demand from MG, c) Hourly selling energy to the MG.

In this subsection, the HEMS is considered with ρ=1. The results for this scenario are shown in Fig. 15, Fig. 16, and Table 13. Fig. 15 displays the Pareto Front of the optimal solutions for this scenario. The results of SoE, load demand, and selling power to MG of MOEMS for marked solution in Fig. 15 with electricity cost (806.363) and PAR (2.487) are shown in Fig. 16. Fig. 16a is shown the SoE of ESS. The required energy for SH and energy demand from MG are shown in Fig. 16b. Fig. 16c depicts the selling energy to MG. Shiftable load scheduling for the marked solution is illustrated in Table 13.

**Table 13:** Shiftable Loads scheduling for case-4 with ρ=1 for marked solution in Fig. 15 with electricity cost equal to 806.363 U.S. cents and PAR equal to 2.487.

Shiftable Loads	Used time slots	Shiftable Loads	Used time slots	Shiftable Loads	Used time slots	Shiftable Loads	Used time slots
Bread Machine	14∼17	Dish Washer	38∼43	Main Air Conditioner	1, 5, 7, 8, 11∼14	Disinfection Cabinet	19, 37, 39, 46∼48
Spin Dryer	34∼37	Music center	34∼37	Living room Air Conditioner	39∼44	EV	5∼8, 12, 13
Cooker Oven	21∼24	Bath heater	45, 45	Workroom Air Conditioner	23, 28, 33, 38	Bedroom Air Conditioner-1	9, 10, 12–14, 16
Coffee Machine	18, 19	Electric kettle	26∼29	Water Heater	21, 22, 31∼44	Bedroom Air Conditioner-2	7∼10, 14, 20
Washing Machine	31∼34	Juicer	37,38	Laptop	29, 39, 40, 34		

#### 5.5.2 ρ=0.8

**Fig. 17:**
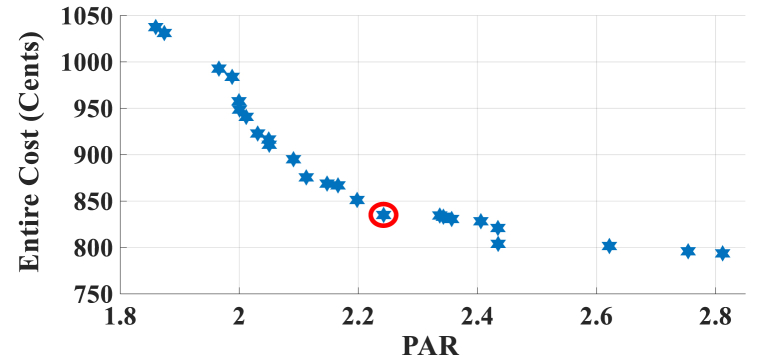
Multi-objective Pareto optimal solution set for case-4 with ρ=0.8.

**Fig. 18:**
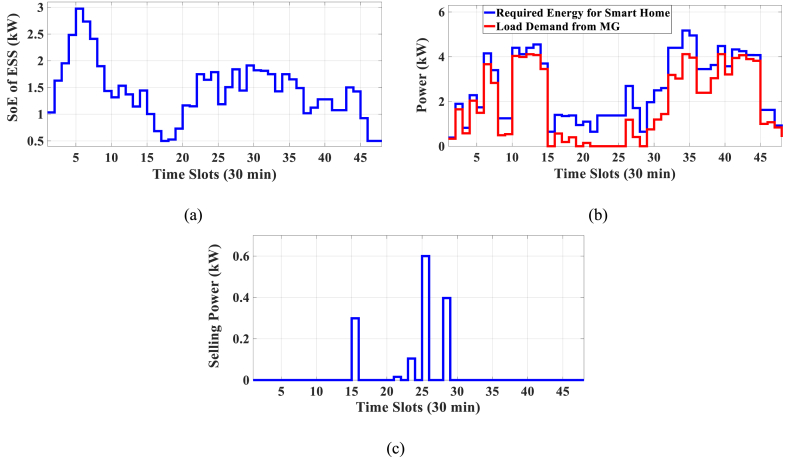
MOEMS for case-4 with ρ=0.8 for marked solution in Fig. 17 with electricity cost equal to 835.076 U.S. cents and PAR equal to 2.242: a) SoE of ESS, b) Hourly load demand from MG, c) Hourly selling energy to the MG.

In this subsection, the HEMS is considered with ρ=0.8. The results for this scenario are shown in Fig. 17, Fig. 18, and Table 14. Fig. 17 displays the Pareto Front of the optimal solutions for this scenario. The results of SoE, load demand, and selling power to MG of MOEMS for marked solution in Fig. 17 with electricity cost equal to 835.076 U.S. cents and PAR equal to 2.242 are shown in Fig. 18. Fig. 18a is shown the SoE of ESS. The required energy for SH and energy demand from MG are shown in Fig. 18b. The selling energy to the MG is shown in Fig. 18c. Shiftable load scheduling for the marked solution is illustrated in Table 14.

**Table 14:** Shiftable Loads scheduling for case-4 with ρ=0.8 for marked solution in Fig. 17 with electricity cost equal to 835.076 U.S. cents and PAR equal to 2.242.

Shiftable Loads	Used time slots	Shiftable Loads	Used time slots	Shiftable Loads	Used time slots	Shiftable Loads	Used time slots
Bread Machine	17∼20	Dish Washer	22∼27	Main Air Conditioner	2, 4, 6, 7, 10, 12–14,	Disinfection Cabinet	32, 33, 45∼48
Spin Dryer	41∼44	Music center	38∼41	Living room Air Conditioner	34∼36, 39, 43, 44	EV	6, 7, 10∼13
Cooker Oven	32∼35	Bath heater	45, 46	Workroom Air Conditioner	37∼40	Bedroom Air Conditioner-1	5, 6, 9–11, 14
Coffee Machine	17, 18	Electric kettle	10∼13	Water Heater	26, 30∼44	Bedroom Air Conditioner-2	8, 11–14, 16
Washing Machine	44∼47	Juicer	17, 18	Laptop	39∼42		

### 5.6 Case-5: Considering the proposed SHEM with costs of RERs

In this case study, the prices of PV, and WT are considered in the electricity cost, and the price of produced energy by WT, and PV are 20 U.S. cents/kWh. Here, it is assumed, that the RERs belong to the SH, hence, the priority is using the produced energy by RERs and ESS, and the required surplus energy is bought from the MG (See Eq. (25)). Also, to consider the cost of produced energy by RERs in entire cost, the cost of generated energy is added to the first objective function (Eq. (28)), and it can be rewritten as Eq. (38):

(38) $$OF_{1}=\sum_{t=1}^{T}\left(P_{SH}\left(t\right)-\rho P_{ESS}^{dis,MG}\right)*Price^{MG}\left(t\right)+\left(P_{PV}\left(t\right)+P_{WT}\left(t\right)\right)*Price^{RER}$$

Where, $Price^{RER}$ is the price of produced energy by RERs (PV, and WT); $P_{PV}\left(t\right)$, and $P_{WT}\left(t\right)$ are the produced power by PV, and WT, respectively.

In this case study, the prices of PV, and WT are considered in the proposed HEMS. The considered system in this case includes PV, WT, and ESS with $Ch_{rate}=0.3$ (kW/Time Slot), and ρ=0.8. As the RERs belong to the SH, the entire cost of generated power is 695.22 U.S. cents for a day (This cost can be changed due to the generated power of RERs varies on different days).

**Fig. 19:**
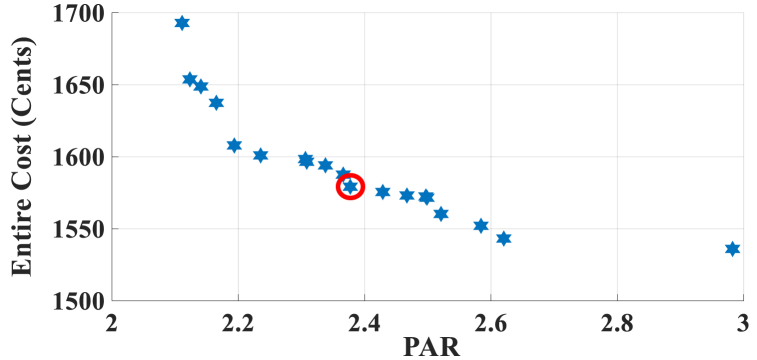
Multi-objective Pareto optimal solution set for case-5.

**Fig. 20:**
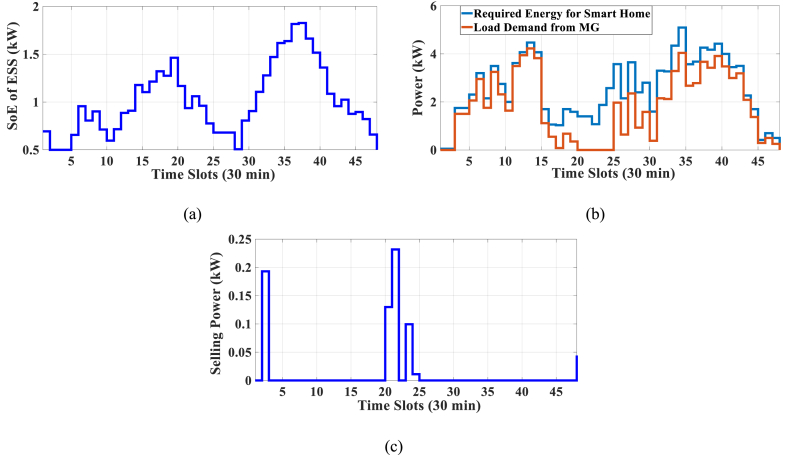
MOEMS for case-5 for marked solution in Fig. 19 with electricity cost equal to 1579.218 U.S. cents and PAR equal to 2.377: a) SoE of ESS, b) Hourly load demand from MG, c) Hourly selling energy to the MG.

The results of case-5 are shown in Fig. 19, Fig. 20, and Table 15. Fig. 19 displays the Pareto Front of the optimal solutions for this case study. As can be seen in Fig. 19, by considering the cost of generated power of RERs, the total cost is increased in comparison to the previous case studies. The results of SoE of ESS, required energy for SH, energy demand from MG, and the selling energy to MG of MOEMS for marked solution in Fig. 19 with electricity cost equal to 1579.218 U.S. cents and PAR equal to 2.377 are shown in Fig. 20. Fig. 20a is shown the SoE of ESS, as can be seen, in high price times, for example, 20, 21, 23–25 time slots, the ESS is discharged to feed the loads and also sales energy to the upstream MG as shown in Fig. 20c. Fig. 20b shows the required energy for SH and energy demand from MG. The sold energy to the MG is depicted in Fig. 20c, as can be seen, the SH is selling energy in 2, 20, 21, 23, and 24 intervals. Shiftable load scheduling for the marked solution is presented in Table 15.

**Table 15:** Shiftable Loads scheduling for case-5 for marked solution in Fig. 19 with electricity cost equal to 1579.218 U.S. cents and PAR equal to 2.377.

Shiftable Loads	Used time slots	Shiftable Loads	Used time slots	Shiftable Loads	Used time slots	Shiftable Loads	Used time slots
Bread Machine	15∼18	Dish Washer	34∼39	Main Air Conditioner	3, 4, 8–11, 13, 14	Disinfection Cabinet	40, 42–44, 46, 47
Spin Dryer	24∼27	Music center	34∼37	Living room Air Conditioner	21, 27, 38∼41	EV	5∼8, 12, 13
Cooker Oven	31∼34	Bath heater	42, 43	Workroom Air Conditioner	23, 27, 28, 37	Bedroom Air Conditioner-1	6, 11∼15
Coffee Machine	13, 14	Electric kettle	9∼12	Water Heater	14, 25, 28, 29, 33∼44	Bedroom Air Conditioner-2	9, 11, 12, 18∼20
Washing Machine	22∼25	Juicer	39, 40	Laptop	23, 33∼35		

### 5.7 Sensitivity analysis

In this section, to show the effectiveness of the proposed method, a sensitivity analysis is presented between the suggested improved BBO (IBBO) and other well-known optimization algorithms such as gray wolf optimizer (GWO), and whale optimization algorithm (WOA). Therefore, in order to have a clear comparison between the methods, based on Eq. (28), and Eq. (31), the total cost function is calculated by Eq. (39):

(39) $$OF=k_{1}*OF_{1}+k_{2}*OF_{2}$$

Where OF is the total objective function, $k_{1}$ and $k_{2}$ are the weight factors that are set to 1 and 50, respectively. The standard deviation and the average of solutions are considered for comparisons between IBBO, GWO, and WOA. 10 independent runs are implemented for each optimization algorithm. The best solution, worst solution, standard deviation, and average are shown in Table 16. As can be seen, the IBBO achieved the first rank in standard deviation and average of solutions in all cases, and then GWO and WOA reached the second and third ranks, respectively. The maximum standard deviation of total objective function between all cases for IBBO, GWO, and WOA are 6.55, 17.22, and 24.87, respectively, which show the performance and superiority of IBBO in finding the best solution in comparisons of GWO, and WOA.

**Table 16:** Sensitivity analysis.

Case Study		Method	Best Solution			Worst Solution			Standard deviation			Average		
			OF	$OF_{1}$	$OF_{2}$	OF	$OF_{1}$	$OF_{2}$	OF	$OF_{1}$	$OF_{2}$	OF	$OF_{1}$	$OF_{2}$
Case-2	Sans RERs	IBBO	1272.31	1168.07	2.08	1279.27	1169.76	2.19	1.94	2.84	0.05	1276.41	1171.50	2.10
		GWO	1278.62	1178.96	1.99	1306.48	1198.44	2.16	8.42	6.79	0.057	1290.7	1186.48	2.08
		WOA	1300.56	1184.40	2.32	1375.33	1258.65	2.33	24.87	25.49	0.12	1326.97	1219.04	2.16
	With RERs	IBBO	884.69	756.45	2.56	902.34	788.20	2.28	4.67	8.00	0.096	892.69	771.39	2.43
		GWO	887.76	770.71	2.34	938.50	820.74	2.32	17.22	17.22	0.14	906.88	786.97	2.40
		WOA	903.81	787.49	2.33	964.16	842.37	2.44	17.60	18.77	0.17	931.94	807.03	2.49
Case-3	$Ch_{rate}=0.6$	IBBO	878.98	747.15	2.64	893.16	755.6	2.76	3.58	7.22	0.13	886.48	760.97	2.51
		GWO	877.83	741.65	2.72	924.63	795.56	2.58	13.30	14.51	0.14	890.58	767.76	2.46
		WOA	910.20	796.48	2.27	942.57	827.15	2.31	9.29	9.66	0.12	927.80	808.69	2.38
Case-4	ρ=0.8	IBBO	874.14	752.44	2.43	898.52	775.97	2.45	6.55	7.76	0.10	888.34	765.27	2.46
		GWO	873.58	753.77	2.40	907.92	789.75	2.36	10.68	9.95	0.14	889.14	766.78	2.45
		WOA	905.65	785.95	2.39	967.28	837.68	2.59	16.76	14.61	0.13	933.28	808.95	2.49

The average of total cost function for the second case study, without RERs for the IBBO, GWO, and WOA are 1276.41, 1290.7, and 1326.97 U.S. cents respectively. It is observed that the average of total cost function value of the IBBO is 14.29 and 50.56 U.S. cents less than the GWO and WOA, respectively. Also, the average of total cost function value for the second case study considering both PV and WT for the IBBO, GWO, and WOA are 892.69, 906.88, and 931.94 U.S. cents, respectively. It is observed that the total cost function value of the IBBO is 14.19 and 39.25 U.S. cents less than the GWO, and WOA, respectively.

By considering the average of the total objective function which is presented in Tables 16 and it can be seen that, by increasing the charging/discharging rate from 0.3 (case-2 with RERs) and 0.6 (kW/Time slot), the total objective function is reduced from 892.69 to 886.48 (total objective function reduced by 0.7 %). Compared to other optimization algorithms, the total cost function value for the third case study (when the ESS charging/discharging rate is 0.6), the average of the total objective function of IBBO, GWO, and WOA are 886.48, 890.58, and 927.80, respectively. It is observed that the total cost function value of the IBBO is 4.1 and 41.32 less than the GWO and WOA, respectively.

For the fourth case study with ρ=0.8, the average of total objective functions of IBBO, GWO, and WOA are 888.34, 889.14, and 933.28, respectively. Hence, the average of total cost function value of the IBBO is 0.8 and 44.94 less than the GWO and WOA, respectively.

## 6 Conclusion

In this paper, a model for optimizing HEMS was presented based on PAR and SH costs. Various kinds of RERs such as PV, WT, and ESS are considered in determining the schedules of home devices, and single- and multi-objective optimizations are performed. Also, the proposed HEMS is considered under different charging/discharging rates and various selling electricity tariff ratios. In addition to enhancing the interaction between SHs and MG, it can facilitate households' active participation in selling energy in DR. Scheduling time slots are set at 30 min, ensuring a small enough interval for flexible and optimum scheduling. According to simulations, ESS and RERs offer more scheduling options and reduce PAR and energy costs more effectively. Further, multi-objective optimization can optimize devices while simultaneously considering energy cost and PAR, ensuring each objective does not sacrifice the advantages of the others and reaching a suitable solution. The maximum standard deviation of total objective function between all cases for IBBO, GWO, and WOA are 6.55, 17.22, and 24.87, respectively, which show the superiority and robustness of IBBO in finding the best solution in comparisons of GWO, and WOA. Also, the results show that the IBBO algorithm has a better performance in comparisons of GWO, and WOA to find the best solution in all case studies.

For the future, we plan to consider the HEMS with more objective functions such as user satisfaction and emissions, also thermal energy will be added to the system to consider both thermal and electrical loads together. Furthermore, deep learning methods can be used to estimate the exact data of renewable energy generation and the required energy of appliances. Also, the uncertainties of electric vehicles and RERs will be regarded in the SHs. Furthermore, cloud-fog computing can be used for an MG with several SHs to perform energy management for each home as edge computing and then transfer the results to the upper layer to reduce computation burdens and increase the systems’ cyber-security.

## CRediT authorship contribution statement

**Moslem Dehghani:** Writing – review & editing, Writing – original draft, Software, Formal analysis, Data curation, Conceptualization, Investigation, Methodology, Resources, Validation, Visualization. **Seyyed Mohammad Bornapour:** Writing – review & editing, Writing – original draft, Validation, Supervision, Software, Resources, Project administration, Methodology, Investigation, Formal analysis, Conceptualization, Data curation, Visualization.

## Data availability statement

Data is contained within the article.

## Ethics approval statement

Review and/or approval by an ethics committee was not needed for this study because this article does not involve any human and behavioural studies, and animal experiments.

## Funding information

This research did not receive any specific grant from funding agencies in the public, commercial, or not-for-profit sectors.

## Declaration of competing interest

The authors declare that they have no known competing financial interests or personal relationships that could have appeared to influence the work reported in this paper.
